# The Influence of Dentition in the Etiology of Epilepsy

**Published:** 1905-01-07

**Authors:** 


### THE INFLUENCE OF DENTITION IN THE ETIOLOGY OF EPILEPSY.

DR. WILLIAM SPRATLING, whose writings on the subject of epilepsy are well known, communicates an article to the *Medical News* of December 10th, in which he strongly upholds the view that there is an important connection between epilepsy and dentition, especially the first dentition. It is never justifiable, he thinks, to regard the convulsions of infancy and early life as harmless manifestations. Numerous infants, it is true, suffer from convulsions which are not repeated in after life. But in no particular instance can future immunity be certainly promised, and therefore, in order to be on the safe side, it is wise to take a serious view; a course which will be all the more necessary if there is any evident liability to a neuropathic inheritance. Dr. Spratling has made an inquiry into the time of life at which convulsions first made their appearance in 3,523 patients in whom epilepsy developed before the end of the twentieth year. The results which he has obtained, and which he has illustrated by charts, are worthy of attention. The greatest number of cases developing in one year was 376, and occurred in the first year of life. All these may be ascribed to congenital causes, to heredity, to birth accidents, and to the first dentition. After the first year there is a continuous decrease through the second, third, fourth, fifth, and sixth years, when 127 cases only out of 3,523 occurred. Then the seventh year (period of second dentition) shows an increase to 168, this increase continuing through the eighth year, the number of cases reaching 174. After this a fluctuating rate is noted until the twelfth year, when the stress of puberty begins to be felt. The four years that cover this developmental period show a large increase in the number of cases, a total of 877 out of 3,523 occurring during these four years. The fourteenth year, which marks the acme of the influence of the period of puberty, stands second in the number of cases for one year periods, there being 258 cases in that year as compared with 376 during the first year. After the epoch of puberty the decline is sharp and continuous, so much so that by the end of the twentieth year the time has passed during which more than eight-tenths of all cases of epilepsy occur.

An analysis of the cases developing in the first year shows that 27 per cent. exhibited signs of epilepsy in the first month of life, while in another 25 per cent. the convulsions first appeared during the sixth month, numbers which are markedly in excess of those for any of the other 10 months.

From these figures, it appears that the first onset of epilepsy most commonly occurs during the first month of life, at puberty, or during the first or second dentitions. The influence of puberty has been generally recognised, but it is doubtful if sufficient attention has previously been given to the influence of dentition, or at any rate to the concurrence of the onset of epilepsy with the eruption of the first or the permanent teeth.

